# A description of externally recorded womb sounds in human subjects during gestation

**DOI:** 10.1371/journal.pone.0197045

**Published:** 2018-05-10

**Authors:** Joanna J. Parga, Robert Daland, Kalpashri Kesavan, Paul M. Macey, Lonnie Zeltzer, Ronald M. Harper

**Affiliations:** 1 Pediatrics, Division of Neonatology, Children’s Hospital of Philadelphia, Philadelphia, Pennsylvania, United States of America; 2 Department of Linguistics, University of California, Los Angeles, California, United States of America; 3 Pediatrics, Division of Neonatology, University of California, Los Angeles, California, United States of America; 4 Neurobiology, Brain Research Institute, University of California, Los Angeles, California, United States of America; 5 Pediatrics, Pediatric Pain and Palliative Care, University of California, Los Angeles, California, United States of America; TNO, NETHERLANDS

## Abstract

**Objective:**

Reducing environmental noise benefits premature infants in neonatal intensive care units (NICU), but excessive reduction may lead to sensory deprivation, compromising development. Instead of minimal noise levels, environments that mimic intrauterine soundscapes may facilitate infant development by providing a sound environment reflecting fetal life. This soundscape may support autonomic and emotional development in preterm infants. We aimed to assess the efficacy and feasibility of external non-invasive recordings in pregnant women, endeavoring to capture intra-abdominal or womb sounds during pregnancy with electronic stethoscopes and build a womb sound library to assess sound trends with gestational development. We also compared these sounds to popular commercial womb sounds marketed to new parents.

**Study design:**

Intra-abdominal sounds from 50 mothers in their second and third trimester (13 to 40 weeks) of pregnancy were recorded for 6 minutes in a quiet clinic room with 4 electronic stethoscopes, placed in the right upper and lower quadrants, and left upper and lower quadrants of the abdomen. These recording were partitioned into 2-minute intervals in three different positions: standing, sitting and lying supine. Maternal and gestational age, Body Mass Index (BMI) and time since last meal were collected during recordings. Recordings were analyzed using long-term average spectral and waveform analysis, and compared to sounds from non-pregnant abdomens and commercially-marketed womb sounds selected for their availability, popularity, and claims they mimic the intrauterine environment.

**Results:**

Maternal sounds shared certain common characteristics, but varied with gestational age. With fetal development, the maternal abdomen filtered high (500–5,000 Hz) and mid-frequency (100–500 Hz) energy bands, but no change appeared in contributions from low-frequency signals (10–100 Hz) with gestational age. Variation appeared between mothers, suggesting a resonant chamber role for intra-abdominal space. Compared to commercially-marketed sounds, womb signals were dominated by bowel sounds, were of lower frequency, and showed more variation in intensity.

**Conclusions:**

High-fidelity intra-abdominal or womb sounds during pregnancy can be recorded non-invasively. Recordings vary with gestational age, and show a predominance of low frequency noise and bowel sounds which are distinct from popular commercial products. Such recordings may be utilized to determine whether sounds influence preterm infant development in the NICU.

## Introduction

Environmental noise has the potential to negatively impact developing auditory, hormonal, and neural systems in premature infants. The concern for consequences of excessive noise has led to environmental health guidelines for neonatal intensive care units (NICUs) that promote low sound levels.[1,2] However, these recommendations were promoted at a time when NICUs were open-units shared by many infants, nursing staff and other health care providers.[3] Currently, NICUs are moving toward designs with primarily private rooms. The combination of low-noise practices and private rooms may potentially lead to relative sensory deprivation.[4] A very low noise environment may be detrimental to a premature infant’s physiological and neural growth.[4]

Sounds found in NICU’s are considered harmful to infants because of the substantial differences between NICU and intra-uterine sound environments.[5,6] Maternal tissues and amniotic fluid buffer high sound pressure levels and high frequency outputs before reaching the fetal ear.[6,7] Those circumstances, coupled with a replacement of bone for air conduction in sound wave propagation, makes the NICU a vastly different acoustical environment from the womb.[6,8] That said, the womb environment is noisy, ranging from 70–90 dB.[8] Intra-uterine acoustics have been examined by inserting hydrophones, or specialized waterproof microphones, in the necks of fetal lambs during gestation.[9] Knowledge of unique uterine sound spectra is supplemented in humans by the insertion of hydrophones into the cervix at the time of delivery.[7,10] Current NICU sound guidelines advocate for no ambient sound exposure in the NICU above 45 to 50 dB at baseline, and over 65 dB in transient spikes. There are no current recommendations on the frequency or composition of sound exposure.[6] However, recorded intra-abdominal sounds in pregnancy show an environment where average decibel levels exceed current NICU sound recommendations by 10 to 30 dB.[9,10]

Womb-like sounds may be a powerful tool in aiding cardiorespiratory stability, pain mitigation, and sleep promotion in infants.[11–15] Instead of simply reducing sound levels, recreating the intra-uterine sound environment may be a means to promote infant development in the NICU.[15] Recreating other *in utero* sensory experiences is beneficial; for example, maternal stimulation and involvement leads to better outcomes for babies born early with practices like kangaroo care.[16] However, before such a possibility could be tested, sound recordings that faithfully assess the intrauterine experience are needed. Technological advances in auscultation may now allow for a broader spectrum of sounds to be recorded and quantified within pregnant abdomens without resorting to invasive measures.[17] If these sounds could be adequately captured, their ability to simulate the intra-uterine environment may aid in understanding the role maternal biological sounds play in development.

This study was designed as a descriptive investigation to assess the efficacy and feasibility of external non-invasive recordings in pregnant women. We endeavored to 1) capture intra-abdominal or womb sounds during pregnancy with electronic stethoscopes; 2) build a womb sound library and assess trends in sounds with gestational development, and 3) compare these sounds to popular commercial womb sounds marketed to new parents.

## Materials and methods

A descriptive study was performed to collect normative data of externally recorded sound profiles around the human uterus during pregnancy. We studied 50 pregnant women and 5 non-pregnant participants outlined in **Table 1**. Women were enrolled from the midwife clinics at the University of California, Los Angeles (UCLA) located in Westwood, California. The UCLA Institutional Review Board approved the study, and informed written consent was obtained from all participants. Inclusion criteria required that the pregnant women be in their second or third trimester of pregnancy as fetal hearing develops between 16 and 24 weeks.[18] Any pregnant woman less than 18 years of age was excluded from study. Study participants were recorded at one study visit and not over time. Recruitment for the study occurred from August 2016 to February 2017.

**Table 1:** Characteristics of pregnant women and non-pregnant women. (N = 55).

	Pregnant Women (50)	Non-Pregnant Women (5)
**Age (Years)**	33 ± 3.8	35 ± 5.8
**Gestational Age (Weeks)**	30 ± 6.7	N/A
**Body Mass Index (BMI)**	27.5 ± 3.7	24.9 ± 6.4
**Time Since Last Meal (Hours)**	2.1 ± 1.2	2.6 ± 2

All recorded sounds were obtained with electronic stethoscopes (*Think labs One*, Centennial, USA). The stethoscopes were attached to a digital recorder (Tascam US-4x4 USB Audio Interface, Japan) that allowed for simultaneous four-point recordings with an electronic stethoscope present on the right upper and lower quadrants and left upper and lower quadrants of the abdomen (S1 Fig). Data were captured on a computer audio interface (Logic Pro X, Cupertino, USA). Recordings were performed in a private clinic room, and mothers were asked to remain silent during the acquisition to exclude maternal voice from analysis. The mothers were recorded between 2pm and 5pm, and lasted a total of 6 minutes, with the mother recorded for 2 minutes standing, 2 minutes sitting, and 2 minutes lying supine. The focus was to capture spontaneous involuntary biological sounds. The sounds recorded in pregnant mothers were compared to four commercially-available sounds that are marketed as mimicking sounds from the womb (labeled #1-#4 for analysis). The commercial womb sounds consisted of sounds found when searching “womb sounds” and selecting those that were highly rated by consumers on Amazon.com.^TM^

Each subject was recorded once in the clinic setting. When listening to the recordings there is a considerable amount of variability within subjects. There was also intra-subject variability, however, the sound profiles were very similar from mother to mother. The study team was able to qualitatively notice major differences in biological sound profiles as the recordings progressed over the 2 minute sound period (S1 File). Two separate examiners listened to all the recording to verify the presence of different types of maternal sounds (fetal and maternal heartbeat, bowel sounds, respiratory sounds), and to determine markers of analysis to compare and contrast the sounds heard.

Multiple structural variables have the potential to modify recorded sounds, including maternal BMI, which varied considerably between subjects. However, we focused on the most major characteristic for change the gestational age of the fetus. While other sound studies have emphasized sound pressure levels, we focused on decomposing sound into spectral and temporal domains. For spectral analysis, we used Long Term Average Spectra (LTAS). The LTAS is defined as the energy density per unit frequency, averaged over time.[19] These frequency ranges were further condensed by averaging across a low band (10-100Hz), mid band (100-500Hz), and high band (500-5000Hz). Raw waveforms were mapped to LTAS using PRAAT (Phonetics Sciences, Amsterdam, The Netherlands), software which is commonly used to analyze speech phonetics.[20] The spectral band data were analyzed with linear mixed-effect regression using the lme4 package in RStudio (RStudio, Boston, USA).[21,22] A separate model was used for each band; each model included gestational age as a fixed effect and a random intercept for mother.

For temporal analysis, we examined discrete events occurring during recordings. Peaks were identified by mapping the waveform to intensities using PRAAT's default settings, and then identifying intensity peaks using a built-in function.

## Results

### Variation within- and across-mothers, compared to non-pregnant abdomens

**Fig 1:**
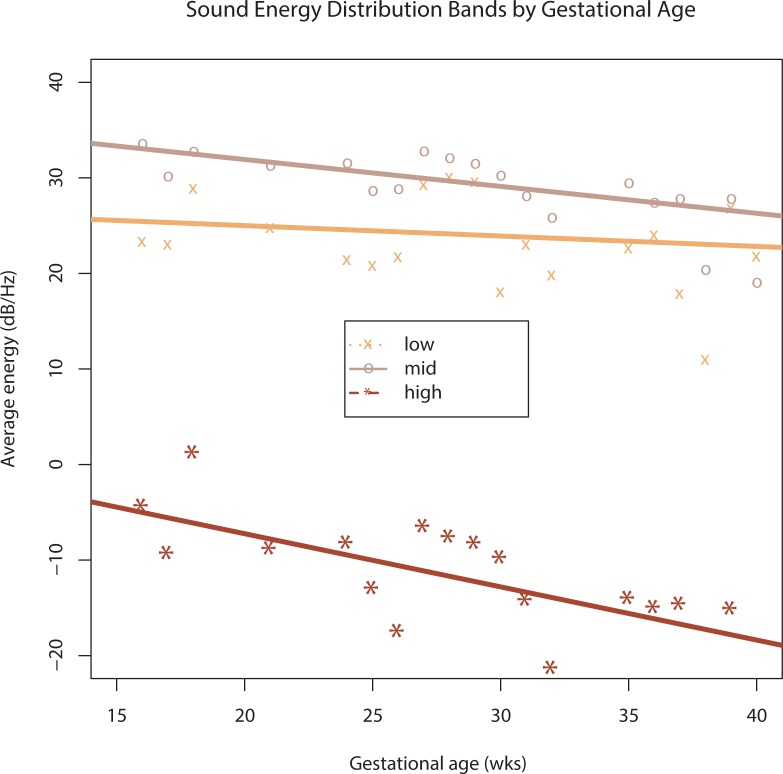
Sound frequency bands across gestation. Distribution of energy across low, mid, and high frequency bands (normalized by bandwidth). Points represent bins by week (*n* = 50).

There was a qualitative perception of similarity of sounds to womb recordings within and across mothers noted by the research team. Principal characteristics included periodicity from the maternal cardiovascular system, and a vacuous low-frequency sound from the bowel that was random in nature. However, there were also significant quantifiable dimensions of variation. A major difference was found in relation to gestational age. To quantify this aspect, we measured the average loudness per unit frequency across the low frequency band (10–100 Hz), mid band (100–500 Hz) and high band (500–5000 Hz). Fig 1 plots these loudness values against gestational age (collapsed within weeks), with trend lines. The linear mixed-effect regression analysis showed that as the abdomen grows while the fetus is developing, it filters more and more energy in the mid (*t* = -3.391, *significant*) and high bands (*t* = -3.417, *significant*), but not in low bands (*t* = -0.717, *not significant*).

**Fig 2:**
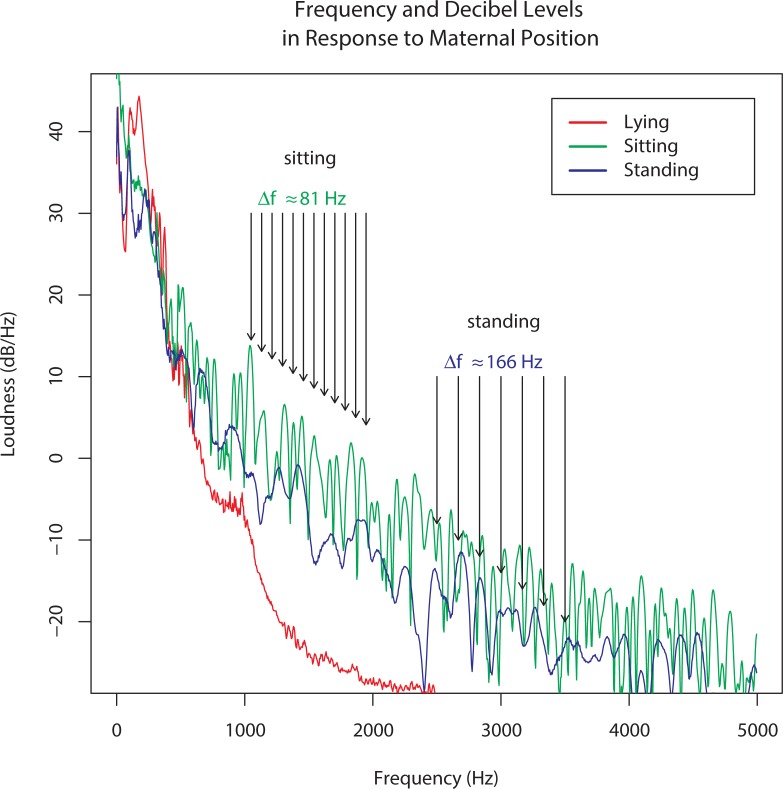
Frequency spectra for a mother recorded in three different poses. Long term average spectra (LTAS) for a single mother in lying, sitting, and standing poses. The regularly spaced peaks in the sitting and standing poses indicate resonance, with varying fundamental frequencies.

In addition to variation with gestational age, within-mother variation emerged. Fig 2 shows the LTAS for one mother in three different positions: sitting, standing, and lying supine. When the mother is lying, higher frequencies were especially damped. However, in the sitting and standing positions, more high frequency sound was present.

Comparing the pregnant womb sounds to those from non-pregnant abdomens, the only observed conclusions were that the non-pregnant abdominal recordings consistently had little intra-subject variability. There was inter-subject variability noted between mothers, particularly on measures assessed with gestational age. Most of the changes in intraabdominal or womb acoustic properties took place in the second and third trimesters with filtering of high frequency sounds.

### Bowel sounds

**Fig 3:**
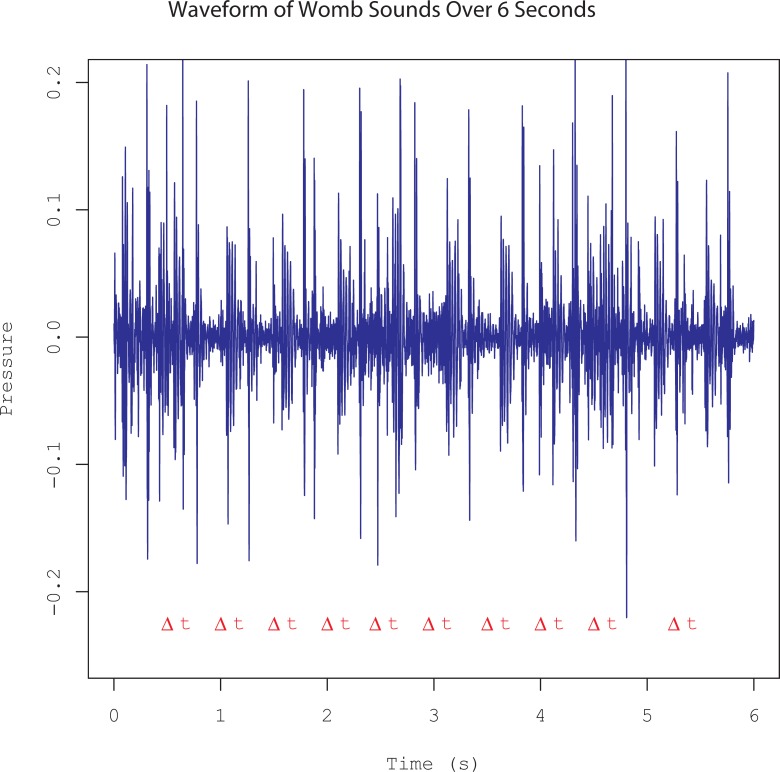
Waveforms from maternal recording noting bowel sound occurrence. Waveforms of 6 seconds of recordings from one mother. Blue indicates recorded sound pressure level, black | marks indicate automatically-detected bowel sounds. Measured time intervals between bowel sounds indicated with red 'At'.

Various biological sounds could be heard in the recordings including maternal heartbeat, fetal heartbeat (distinguished from maternal heartbeat by the rate), respiratory sounds, and bowel sounds. Bowel sounds were the most prominent acoustic feature of the womb recordings, and were distinguished by the presence of frequent, loud popping sounds reminiscent of gas movement. Bowel sounds as loud popping sounds were evident on the waveform as high-amplitude transient events. It was, therefore, possible to automatically detect a large proportion of bowel sounds by identifying intensity peaks. Using PRAAT, loudness peaks which exceeded the 90^th^ percentile of all loudness values were automatically classified as bowel sound events. Fig 3 shows 6 seconds of waveforms from one mother, with the automatically-identified bowel sounds marked below using the '|' symbol. The intervals between successive bowel sounds were collected. In the aggregate, the intervals between bowel sounds followed a Poisson distribution with parameter *k*≈0.2s, i.e., bowel sounds had a constant chance of occurrence per unit time, and there were, on average, 5 "loud" bowel sounds per second.

**Fig 4:**
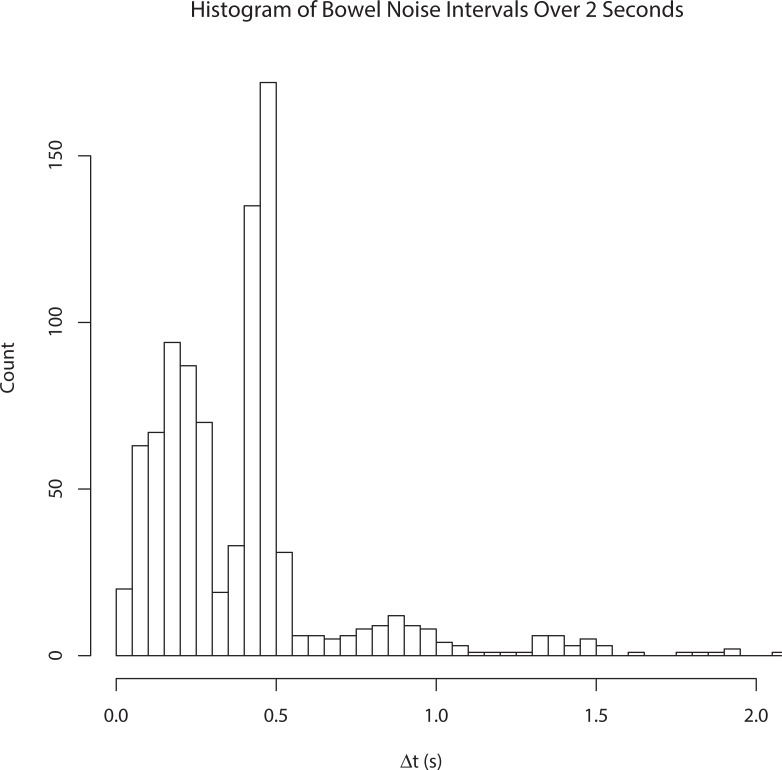
Histogram of bowel sound occurrences. Histogram of time intervals between successive (measured) bowel sounds. Weak rhythmicity evident from primary mode at ≈500ms, secondary mode at ≈250ms.

Fig 4 is a histogram of the intervals between bowel sounds from one mother in the supine position. As evident from the histogram, the distribution is 'rhythmic', with a primary mode at about 0.5s, a secondary mode at ≈0.25s, and additional (much less prevalent) modes at longer intervals. Thus, when a bowel sound occurred, another bowel sound was maximally likely to occur about a half-second later, or about a quarter second later, and occasionally, a longer period before the next bowel sound. The bowel sound distribution was qualitatively the same in non-pregnant abdomens.

Of note, time of last meal was collected for all participants. These values did not appear to affect the timing or duration of when bowel sounds occurred. No other measures of maternal gastrointestinal function were studied (i.e. reflux).

### Comparison with commercially-available womb sounds

**Fig 5:**
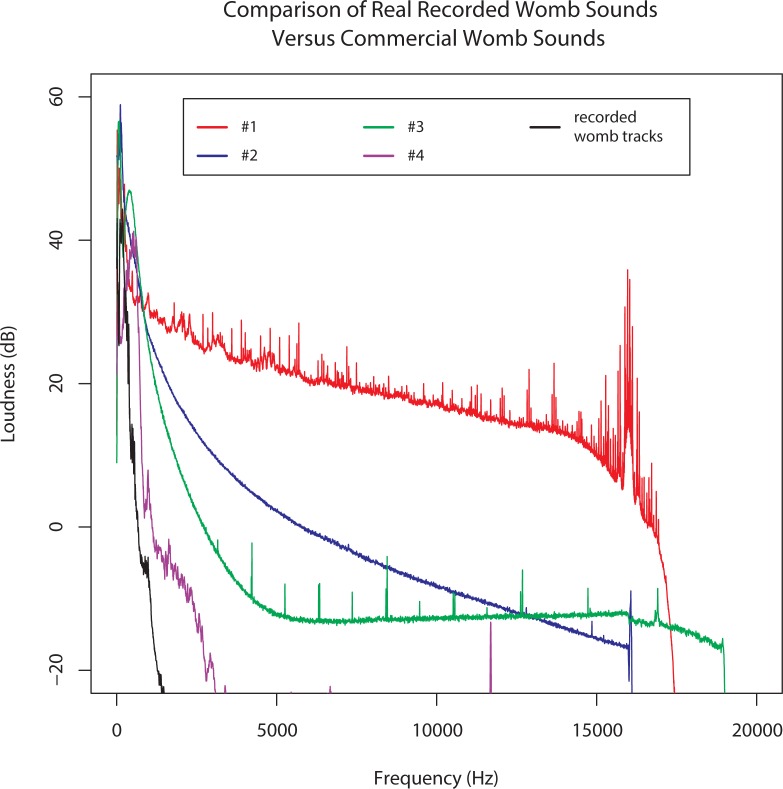
Maternal recordings compared to commercial sounds. Long-Term Average Spectrum (LTAS) for commercial womb sounds and one mother from the study. Only #4 has a similar balance of energy at low versus high frequencies.

**Fig 6:**
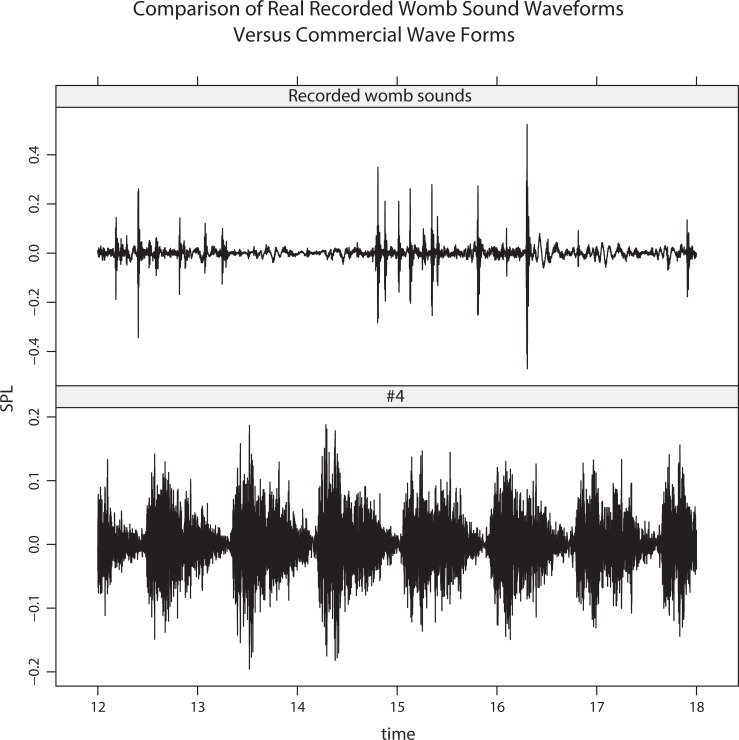
Comparison of waveforms between maternal recordings and commercial sounds. Waveforms (6 s) from a mother (top) and commercial sound #4 (bottom). The pulses in the mother are irregularly occurring bowel sounds; whereas, commercial sound #4 consists of periodic, broadband noise beats.

A salient question is how commercially-available womb sounds compared to our recordings. This comparison was made because these sounds are often used in NICUs and by parents without a good comparison to what real womb sounds are, and with little research on what physiologic effects they exert on infants. To explore this issue, we obtained popular commercial womb sounds available on Amazon.com. Two minutes of data were extracted from four commercial sounds (labeled #1–4). Sound #1 came from “Natural Womb Sounds” by Joe Baker who obtained the sounds nine years ago from a mixture of recordings with electronic and conventional stethoscopes. Sound #2 was a track entitled “Womb Sounds” from the website Amazon.com. Sound #3 was a track entitled “60 Minutes Womb Sounds” also from Amazon.com. Lastly Sound #4 was a track entitled “Mellow” on the *Happiest Baby on the Block Soothing White Noise Sleep Sounds* album. Qualitatively, those samples sounded different from each other, and from our maternal recordings. A common perception was that the commercial sounds mainly consisted of broadband noise (varying in spectrum); in #2, an audible heartbeat is superimposed (approx. 70bpm), and in #4, there is a 'beat' (triangle-shaped amplitude modulation, also about 70bpm). Fig 5 shows the LTAS for all four commercial sounds, together with maternal recordings. Fig 6 shows the waveform for 6 seconds from the same mother, and 6 seconds of commercial sound #4 (where 6 beats are visible).

Another salient difference was that the commercial sounds do not include 'pops' from bowel sounds, whereas such bowel sounds are the loudest feature in the womb recordings. This aspect is illustrated in Fig 6: the bowel sounds are evident as the sharp 'spikes' in sound pressure level.

## Discussion

An earlier study demonstrated the potential for appropriate auditory stimulation to mediate particular autonomic developmental changes.[15] The objective here was to determine the nature of sound exposure to the fetus during development that would presumably act as a calming influence, and could support autonomic and respiratory development. The potential to adequately assess the nature of sound exposure demonstrated here is essential to ensure appropriate exposure in the NICU for acoustical support for the neonate.

We show it is possible to externally record high-fidelity intra-abdominal or womb sounds throughout the 2^nd^ and 3^rd^ trimesters of pregnancy, and that quantifiable variation in recordings is present. In comparison to commercially available womb sounds, maternal recordings were noticeably less regular in spectral variation across time, and contained considerably less energy in the high frequency band ('whispery'). A major source of this variation was the presence of bowel sounds, in which amplitude dominated those from other sources (such as the maternal heartbeat). On average, bowel sounds occurred every 200ms, although there was considerable variation within and between mothers, both in regularity and intensity. An additional source of variation was gestational age–mid and high frequencies become quieter as the womb grows, an outcome possibly arising from distance of the recording site from the viscera. The viscera likely generate less noise secondary to displacement, and possibly from the bowel slowing effects of progesterone, or alternatively, the uterus filters the high frequency sound surrounding the fetus. Non-pregnant abdomens showed similar bowel sound patterns; however, there were few differences between the non-pregnant recordings, while the maternal recordings showed consider variation when accounting for gestational age.

Comparing our recorded sounds to intra-uterine recordings from previous studies showed similarities. In utero, the fetus is subjected to auditory stimuli from the mother’s heartbeat, arterial blood flow, respiratory and digestive sounds.[6] These endogenous sounds have been measured as high as 70 to 90 dB, levels which are outside current NICU recommendations for environmental stimuli.[1] The presence of maternal tissues and amniotic fluid do not appear to muffle external low pitch noises;[23] however, high pitch noises are significantly dampened, with attenuated sound pressure levels of 10 to 25dB.[24] These findings are reinforced by our recordings which show a relatively similar amount of input from low frequency energy bands and a significant drop in high- and mid-frequency energy bands with increasing gestational age.

One of the more interesting findings was that sounds from the abdomens of pregnant women appear to have regularly occurring peaks and valleys indicating *resonance*; the amplification of energy at the fundamental frequency and integer multiples arising from constructive interference as sound waves reflect inside the abdomen. This pattern changes with maternal position, because the dimensions of the womb vary with positioning. The ability to detect resonance is evolutionarily important, e.g. for sound localization in mammals generally, and for speech perception in humans in particular.[25] Previous research has shown the acquisition of speech perception begins in fetal life,[26] and that disruptions in normal fetal development (ie. intra-uterine growth restriction of a fetus) can negatively influence language development.[27] Additionally, infants in the womb respond to the native language of their mother differently than to a foreign language.[28] These findings demonstrate a sophistication in the fetuses ability to discriminate sounds, and highlight the importance of exploring the womb soundscapes influence on the developing brain.

While we did not study the contributions of maternal voice to development, the implications of these findings for fetal development rest with the potential for sound characteristics such as frequency, time-variation and intensity to influence neural, and especially autonomic development postnatally. The assumption is that the long exposure to fetal sounds exercise a “comforting” and perhaps supportive influence on at least autonomic patterning, an assumption based on calming influences exerted by music and other rhythmic sounds in later life. We describe here that the history of sounds to which the fetus is exposed is weighted to low frequencies, with frequent episodic sounds, such as “pops” of bowel activity, and maternal cardiac sources. Those characteristics should drive development of external sound sources to assist supportive auditory input post-natally, and represent characteristics currently not found in common commercial devices. Moreover, the findings suggest that tracking womb sounds with development represents a moving target, and that determination of optimal post-natal sound exposure should consider where in that gestational time course patterns would provide the most supportive environment for the neonate. Such a determination will require more extensive evaluation of post-natal sound influences on autonomic and breathing functions. The extensive variability between mothers suggests that, once the most time-advantageous gestational sound patterns are established to optimize influences on post-natal cardiovascular and respiratory regulation, recordings from the infant’s mother may be most appropriate, rather than standard recordings.

## Limitations

The validity of externally-placed microphones for recordings has not been examined; with enhanced technology, our study aims to begin validating non-invasive external sound capture in humans. We attempted to reduce the effects of exogenous sounds by recording in a quiet clinic room. However, the contributions of external noises should be considered in future studies. In particular, maternal voice should be explored, because, unlike other external noise, maternal vocalization is likely amplified as it enters the uterus by approximately 5dB, reaching levels of 60-75dB.[29] Additionally, we did not measure the maternal abdominal fat pad with calipers or attempt to determine if the recording of sound through the fat, muscle, or skin altered sound qualities. We attempted to assess BMI contributions, but this measure was not associated with differences in sound characteristic measures. These factors can be considered in future studies by controlling for distortion of sound passage through various tissues. While we found changes in the frequency of sound exposure across gestational ages, it would also be informative to record one mother throughout her pregnancy, thus highlighting the influences of a changing individual womb environment on sound exposure to the fetus. Additionally, recording non-pregnant abdomens over time would allow investigators to more adequately compare sounds of pregnant and non-pregnant women.

## Conclusions

It is possible to non-invasively record intra-abdominal or womb sounds during pregnancy with electronic stethoscopes. The recorded sounds show objective and subjective differences when compared across gestational ages and against commercially-available womb sounds. The womb or pregnant abdomen appears to be a resonant chamber which allows for the dissemination of low frequency sounds throughout gestation, with a predominance in bowel noise as a maternal biological stimulus. The availability of this approach to recording womb sounds allows for research into how soundscapes influence development, especially with respect to premature infants in the neonatal intensive care unit who are separated from the womb early. Identification of sound patterns and standardization of the analysis has the potential to improve infant comfort and hospital care by informing clinicians on the optimum NICU sound environment.

## Supporting information

S1 Fig Picture of recording device. This is a photograph of the recording device set up in the clinic where recordings were obtained for the study.
(JPG)

S1 File Recordings of maternal sounds. This is a sound file of a maternal recording that was obtained during this study.
(MP3)
