# Supplementary figures and images for: A description of externally recorded womb sounds in human subjects during gestation
Source: PLoS One. 2018 May 10;13(5):e0197045. doi: 10.1371/journal.pone.0197045 (PMC5944959; doi:10.1371/journal.pone.0197045)

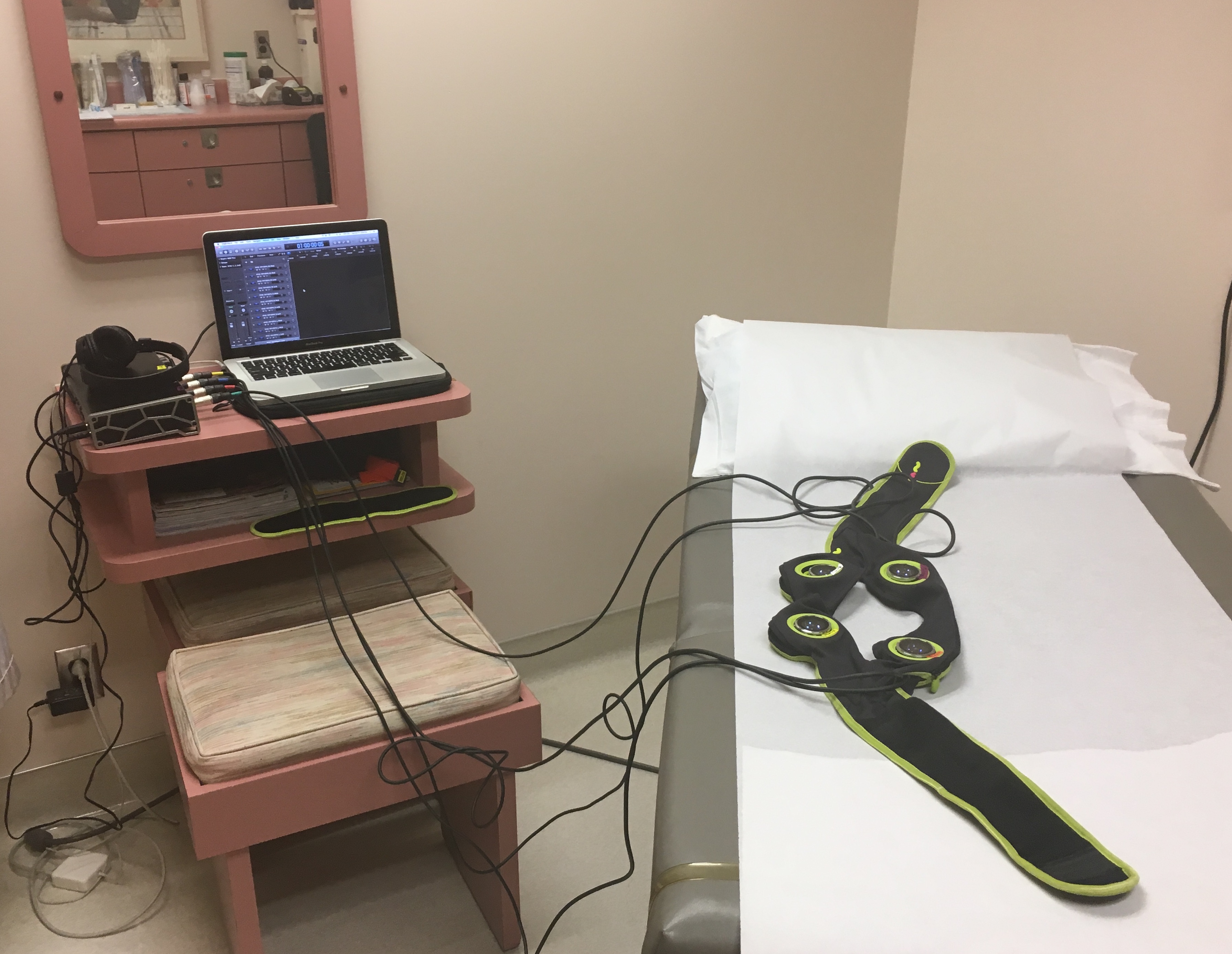

Supplement: S1 Fig — This is a photograph of the recording device set up in the clinic where recordings were obtained for the study. (JPG) [file pone.0197045.s001.JPG]
